# The role of sex differences in cardiovascular, metabolic, and immune functions in health and disease: a review for “Sex Differences in Health Awareness Day”

**DOI:** 10.1186/s13293-025-00714-7

**Published:** 2025-05-13

**Authors:** Lana McClements, Alexandra Kautzky-Willer, Georgios Kararigas, Sofia B. Ahmed, John N. Stallone

**Affiliations:** 1 School of Life Sciences, Faculty of Science, University of Technology Sydney, Sydney, NSW Australia; 2 Division of Endocrinology and Metabolism, Gender Medicine Unit, Medical University of Vienna, Vienna, Austria; 3 Department of Physiology, Faculty of Medicine, University of Iceland, Reykjavik, Iceland; 4 Department of Medicine, Faculty of Medicine and Dentistry, University of Alberta, Edmonton, AB Canada; 5 Department of Veterinary Physiology & Pharmacology, College of Veterinary Medicine, Texas A&M University, College Station, TX 77843-4466 USA

## Abstract

Sexual dimorphism is a fundamental characteristic of the anatomy and physiology of animals and humans, yet biomedical research has largely ignored these phenomena in the study of health and disease, despite early studies in the eighteenth and nineteenth centuries that demonstrated the importance of sex differences. With the explosive growth of biomedical research following World War II, especially in the 1970s through the 1990s, preclinical and clinical studies led to a greater recognition of sex differences in physiological function, particularly the significant disparities in the incidence of and mortality from cardiovascular diseases, which generally occur more frequently in men than in premenopausal women. There is a growing awareness that metabolic and immune dysfunction are intimately tied to the development of cardiovascular diseases. Thus, this review article focuses on sexual dimorphism in cardiovascular, metabolic, and immune function in health and disease, and was prepared for the journal *Biology of Sex Differences* as part of its recognition of “Sex Differences in Health Awareness Day.” This article clearly reveals the striking importance of sex differences in cardiovascular, metabolic, and immune system functions in health and in the pathogenesis of disease processes, which likely involve a combination of effects of the sex chromosomes as well as the gonadal steroid hormones. In the developing fetus, fetal sex clearly influences the pathogenesis of the hypertensive diseases of pregnancy, and sex differences in the effects of the fetus continue beyond pregnancy and appear to influence the future risk of maternal cardiometabolic diseases. Similarly, there is strong evidence of many clinically-relevant sexually dimorphic characteristics of obesity and type 2 diabetes mellitus which appear to involve both chromosomal and humoral effects, although the underlying pathophysiological mechanisms are poorly understood. The gonadal steroid hormones (both androgens and estrogens) are known to exert important effects on the regulation of intermediary metabolism; however, recent studies reveal the emerging importance of these hormones in the regulation of inflammation. For example, menopausal declines in estrogen are associated with increases in inflammatory markers and the development of heart failure in women. Similar effects on inflammatory function may also occur in men with progressive age-dependent declines in testosterone. Declines in androgen levels in men are also associated with detrimental effects on cardiovascular and metabolic function, especially the development of metabolic syndrome and type 2 diabetes, important risk factors for cardiovascular disease. Interestingly, pathophysiological increases in the normally lower testosterone levels in women are associated with the same detrimental effects on cardiovascular and metabolic function, revealing striking bi-directional sex differences in the effects of the androgens. Finally, it is increasingly apparent that the kidney plays an important role in the regulation of sex steroid hormone levels, and the declines in both estrogen and testosterone that occur with chronic kidney disease appear to play an important role in the linkage between chronic kidney disease and the development of cardiovascular disease. In conclusion. It is clear that sex differences in cardiovascular, metabolic, and immune function play important roles in health and in the pathogenesis of disease. Elucidation of the chromosomal and humoral mechanisms underlying sexual dimorphism in physiological functions will play important roles in the future development of age- and sex-specific prevention and pharmacotherapy of disease processes.

## A. Introduction

### John N. Stallone, PhD, FAPS

**Fig. 1:**
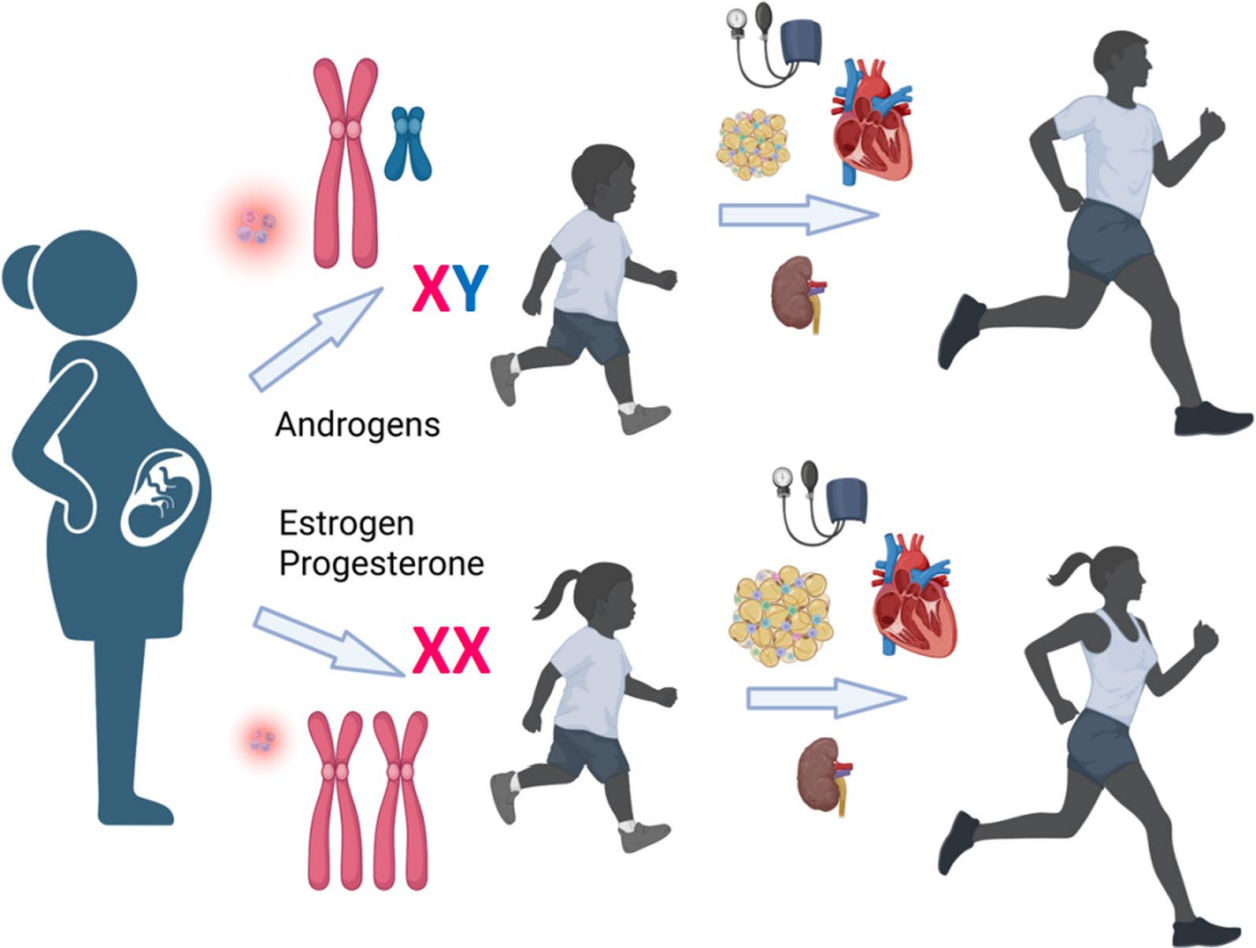
The impact of sex differences begins in utero when chromosomal differences and higher inflammatory environment of the male fetus play a key role. These sex differences continue through childhood and increase with the appearance of the gonadal steroid sex hormones at puberty and into adulthood, with the female sex being more prone to increased adiposity and sex differences in cardiometabolic disease phenotypes and outcomes with increasing age

Sexual dimorphism is a fundamental characteristic of the anatomy and physiology of animals and humans, yet biomedical research has largely ignored these phenomena in the study of health and disease, despite very early studies that demonstrated the importance of these sex differences. For example, the testes in roosters (but not hens) were associated with humorally-mediated effects on the anatomy, behavior and reproductive function in the eighteenth and nineteenth century studies of Hunter and Berthold [1, 2]. Similarly, associations between androgen excess and diabetes, obesity, and infertility in women (but not men) have been known since the report of “diabetes in bearded women” by Achard and Thiers in 1921 [3]. Subsequently, a link between obesity and the triad of polycystic ovaries, hirsutism, and oligo/amenorrhea was first reported in 1935 as the Stein-Leventhal Syndrome, which was later renamed polycystic ovary syndrome [4]. Collectively, these early studies established sex differences in the effects of androgens, which appeared to be beneficial in males, but deleterious in females; however, these sexual dimorphisms in physiological function were largely ignored until the explosive growth of biomedical research in the 1970s through 1990s. These preclinical and clinical studies led to a greater recognition of sex differences in physiological function, particularly the significant disparities in the incidence of and mortality from cardiovascular disease, which occur more frequently in men than in premenopausal women (Fig. 1) [5–10].

What factor(s) underlie(s) the sex differences in form and function observed in animals and humans and how do sex and gender differ? Sex, as defined by the Institute of Medicine [11], is “being male or female, according to reproductive organs and the functions assigned by the chromosomal complement (*i.e.,* XX for female and XY for male)” and the effects of the gonadal steroid hormones. Clearly, sex is a biological characteristic that is dichotomous in nature in the physiological state. In contrast, gender is a psychosocial construct that is a continuous variable and includes factors such as age, behavior, culture, ethnicity, and education. It should be noted that there are congenital abnormalities (DSD: Disorders of Sex Development) in which the individual may exhibit a combination of male and female characteristics. From a biological and experimental standpoint then, every cell has a sex, as determined by the complement of the sex chromosomes and in the intact organism, by the effects of the gonadal steroid hormones. It is not surprising then, to see sexual dimorphisms in cardiovascular, metabolic, and immune function which are present in health as well as disease states.

Although human epidemiological studies on cardiovascular disease in the 1980s led to an increased awareness of sex differences in cardiovascular and metabolic function, it was not until the passage of the National Institutes of Health (NIH) Revitalization Act of 1993 that federally-funded research was mandated to include women in human clinical trials. These human clinical and epidemiological studies led to an increase in preclinical animal studies that that identified sexual dimorphism in systemic blood pressure and isolated vascular function in normal and hypertensive animals [12–16]. As a result of this growing awareness of sex differences in cardiovascular and metabolic health and disease, the Institute of Medicine issued a landmark report in 2001 entitled “Exploring the Biological Contribution of Sex”, which concluded that sex matters in all aspects of cellular function and physiology from “womb to tomb” [11]. In May, 2014, nearly 20 years after the passage of the NIH Revitalization Act that required inclusion of women in clinical research, Francis S. Collins (NIH Director) and Janine A. Clayton (Director of the Office of Research on Women’s Health) announced the intention of NIH to address over-reliance on male cells and animals in preclinical biomedical research [17]. This decision arose from the growing number of studies reporting sex differences in physiological function and the realization that over-use of male cells and animals in preclinical studies could bias their translation to improvements in human health and disease, resulting in adverse effects on women’s health. Thus, taking sex of research animals into account as an important biological variable could improve the reproducibility and value of research results [18–20]. Following this decision, all future applications to NIH were required to include plans to balance the use of male and female cells and animals in all preclinical studies (except for rigorously defined exceptions). While this requirement led to an increase in preclinical studies revealing sex differences in cardiovascular, metabolic, and immune function highly relevant to health and the pathogenesis of disease, the number of published studies examining these differences remains relatively small.

Despite these forward strides in preclinical research, translation to improvements in clinical care still lags behind. For example, cardiovascular diseases in women are still underdiagnosed, undertreated, and understudied [21] and as a result, women die from coronary artery disease at twice the rate of men [22]. There is growing awareness that metabolic and immune dysfunction are intimately tied to the development of cardiovascular diseases [23]. Thus, in this review article prepared for the journal *Biology of Sex Differences* as part of its recognition of “Sex Differences in Health Awareness Day”, the breadth and depth of sexual dimorphism in cardiovascular, metabolic, and immune function and their roles in health and the pathogenesis of disease will be discussed with the goal of increasing reader awareness of the importance of biological sex in the incidence of and mortality from cardiovascular, metabolic, and immune diseases.

## B. The contribution of fetal sex to cardio-metabolic complications in pregnancy

### Lana McClements, PhD, MPharm

#### Characteristics of hypertensive disorders in pregnancy

Hypertensive disorders of pregnancy (HDP) affect 5–10% of pregnancies and are among the leading causes of maternal and fetal morbidity and mortality worldwide [24]. They encompass a spectrum of conditions, including chronic hypertension (HT), white coat HT, gestational HT, preeclampsia, and superimposed preeclampsia (and related disorders: eclampsia and HELLP [haemolysis, elevated liver enzymes, and low platelets] syndrome) [25, 26]. Chronic HT is detected either prior to pregnancy or before 20 weeks of gestation whereas gestational HT and preeclampsia are diagnosed from 20 weeks of gestation. Chronic HT can lead to preeclampsia, condition referred to superimposed preeclampsia [27]. White coat HT is defined as elevated blood pressure (BP) in clinical settings, in the presence of a healthcare professional, while maintaining normal BP in non-clinical settings [26]. However, white coat HT early in pregnancy is reported to progress to gestational HT and in 8% of cases preeclampsia [28]. Gestational HT is considered a new onset or de novo HT (sustained systolic BP ≥ 140 mmHg and diastolic BP ≥ 90 mmHg) in pregnancy without the presence of proteinuria or other type of organ damage typical for preeclampsia (*e.g.* neurological complications, pulmonary oedema, haematological complications, liver dysfunction, or acute kidney injury) [29]. It is expected that BP in pregnant individuals diagnosed with gestational HT will return to normal within three months post-partum [26] Gestational HT can progress to preeclampsia especially in those individuals diagnosed early in pregnancy [30]. Although eclampsia (characterised by seizures) and HELLP syndrome are distinct disorders, these pregnancy complications often occur as a result of poorly managed preeclampsia. HELLP syndrome affects 0.5–0.9% of pregnancies and it is associated with placental dysfunction; it can lead to acute renal and liver failure, disseminated intravascular coagulopathy, and pulmonary oedema, requiring preterm birth in the vast majority of the cases (> 70%) [26]. Eclampsia is characterized by the onset of one or more seizures in individuals with preeclampsia. It remains a significant concern in developing countries, responsible for an estimated 50,000 deaths annually, accounting for around 10% of direct maternal fatalities [24].

HDP can lead to severe short-term and long-term complications for both the mother and fetus. The highest number of deaths due to HDP has been recorded in Latin America and Caribbean [24]. In the short term, maternal complications include preterm labour, placental abruption, pulmonary oedema, renal failure, and eclampsia, which can result in seizures and life-threatening complications. For the fetus, restricted placental blood flow increases the risk of intrauterine growth restriction (IUGR), preterm birth, neonatal intensive care unit admission, and perinatal mortality [31, 32].

Long-term complications extend well beyond pregnancy. Women with a history of HDP are at a significantly higher risk of developing chronic HT, cardiovascular disease, and stroke later in life. Overlapping biomarkers and pathogenic pathways have been identified between preeclampsia, HT, and heart failure with preserved ejection fraction [33]. In the period of the first ten years following HDP, individuals have three- to ten-fold increased risk of developing HT [34]. Additionally, they have an increased likelihood of recurrent hypertensive complications in subsequent pregnancies. Offspring exposed to maternal HT in utero may also face long-term health risks, including a higher predisposition to metabolic syndrome, HT, and cardiovascular disease in adulthood [35, 36]. These complications highlight the importance of continued monitoring and preventive care for both mothers and their children following HDP.

#### Pathogenesis of preeclampsia

Amongst HDP, preeclampsia remains the leading cause of maternal and fetal morbidity and mortality worldwide [32]. Preeclampsia is a heterogenous and multifactorial disorder that includes different phenotypes based on the gestational age of diagnosis: early-onset preeclampsia (< 34 weeks of gestation), late-onset preeclampsia (≥ 34 weeks of gestations), and post-partum preeclampsia (up to 6 weeks post-delivery). Preterm and term preeclampsia are also used when referring to delivery before 37 weeks and from 37 weeks of gestation, respectively [26]. Early-onset preeclampsia has features of placental insufficiency due to inadequate spiral uterine artery remodelling by fetal trophoblast cells leading to poor villous development whereas late-onset preeclampsia has normal placentation with features of overcrowded villous space leading to increased feto-placental demand [32, 37]. In the second stage of preeclampsia development, both phenotypes are characterised by angiogenic imbalance, endothelial dysfunction, oxidative stress, and inflammation [38]. Women with pre-gestational type I or II diabetes mellitus or gestational diabetes mellitus have increased risk of developing preeclampsia, which is linked to pre-existing hyperglycaemia-induced endothelial dysfunction, increased inflammation, and oxidative stress [39].

The largest meta-analysis to date including over ten million participants has shown that individuals affected by preeclampsia in pregnancy have over two-fold increased risk of composite adverse cardiovascular outcomes (odds ratio [OR], 2.05 (95% CI, 1.9–2.3) compared to individuals with normal pregnancies. In this group, the risk of death due to cardiovascular disease was reported to be more than two-fold ([OR] 2.18 (95% CI 1.79–2.66), the risk of renal impairment over three-fold ([OR] 3.35, 95% CI 2.25–5.00), and four-fold higher risk of metabolic syndrome ([OR] 4.05, 95% CI 2.42–6.77) [26, 40]. Interestingly, individuals who suffered early-onset preeclampsia compared to late-onset preeclampsia, in pregnancy, had ~ 1.5–1.7 fold higher risk of composite adverse cardiovascular outcome, renal impairment, and metabolic syndrome and over five-fold increased risk of major cardiovascular events [40]. 

#### Fetal sex and pregnancy

During pregnancy, a tightly regulated balance is maintained between the maternal innate immune cells and placental cells towards a tolerance of a semi-allogeneic fetus and placenta [41]. This is regulated by restricted expression of class Ia and class II human leukocyte antigens (HLAs) in trophoblast cells [42]. When the balance is disrupted and innate immune cells are inappropriately activated, inadequate function of the trophoblasts cells and placentation follow, causing the development of preeclampsia, more commonly early-onset preeclampsia. This process is characterised by elevated inflammation where pro-inflammatory factors including tumour necrosis factor (TNF)-α, interleukin (IL)-6 and IL-1β are increased and anti-inflammatory factors, IL-10, reduced [43].

Fetal sex differences play an important role in pregnancy maintenance and outcomes. For example, male fetuses tend to be larger at 20 weeks of gestation and beyond and have worse birth outcomes than female fetuses [44]. Interestingly, male offspring also exhibit significant differences in body composition, with higher percentages of both fat and lean mass, which appear to be more susceptible to the maternal influence of elevated body mass index and weight gain during pregnancy [45]. Furthermore, the male fetus exhibits a more pro-inflammatory immune response throughout gestation. A study comparing pro-inflammatory and pro-angiogenic factors between individuals carrying male *vs*. female fetuses from uncomplicated pregnancies demonstrated higher concentrations of granulocyte colony-stimulating factor (G-CSF), IL-12p70, IL-21, IL-33, placental growth factor (PlGF), and vascular endothelial growth factor (VEGF)-A in male fetuses across the gestation [46]. The presence of a female fetus was associated with higher systemic concentrations of regulatory cytokines including IL-5, IL-9, IL-17, and IL-25, whereas no differences were observed in the post-partum measurements between male and female offspring in any of these analytes [46]. A male fetus appears to be at a higher risk of infection, preterm birth, and fetal mortality [47]. Pregnant individuals carrying a male fetus appear exhibit an increased risk of pregnancy complications including gestational diabetes, fetal macrosomia, premature rupture of membranes, Caesarian section and other birth complications [48].

The mechanisms underlying the aforementioned sex differences are likely related to sex chromosomes (XX *vs.* XY) and sex steroid hormones (androgens, progesterone and estrogens), which are produced by both sexes however in varying amounts; circulating testosterone in females is 10–15% that of male whereas estrogen in male is 10–30% that of male [49]. During pregnancy, there is a transiently higher production of testosterone at the beginning of the second trimester which can influence the sex-specific pregnancy outcomes [50]. No sex differences in estriol and estradiol levels have been detected between male and female fetuses [51]. Furthermore, in estrogen-deficient mice no differences were found in placental and fetal growth suggesting estrogen is not the underlying mechanism of sex differences in pregnancy [52]. Nevertheless, estradiol has important roles in angiogenesis, placental development and vascular adaptations in pregnancy which are processes aberrantly regulated in preeclampsia in associated with lower maternal circulating estradiol [53]. 

#### Fetal sex and HDPs

Although there is conflicting evidence related to the impact of fetal sex on the risk of HDP including preeclampsia, some sex differences have been reported [54]. As stated above, a male fetus can increase the maternal risk of developing gestational diabetes which, subsequently, is a risk factor for preeclampsia [55]. This association between the presence of a male fetus and gestational diabetes has been attributed to impaired maternal β-cell function and higher blood glucose, suggesting that male sex can impact glucose metabolism adversely in pregnancy [56] .

Interestingly, a meta-analysis including 11 studies with 219,575 independent live-born singleton pregnancies, demonstrated that the influence of fetal sex is dependent on the gestational age, where a female fetus was associated with a higher incidence of early-onset and preterm preeclampsia, whereas no sex differences were reported in the incidence of term preeclampsia. Similarly, another study reported the importance of fetal sex in uteroplacental and cardiovascular adaptation across gestation; thus, uterine artery Doppler showed higher pulsatility index and notching in pregnant individuals with a male fetus in the second and third trimester, indicating the presence of vascular resistance. In a subgroup analysis, the presence of a female fetus in pregnant individuals with pre-eclampsia, fetal growth restriction and/or spontaneous preterm birth (collective referred to as a placental syndrome), was associated with higher diastolic BP in the first trimester. Nevertheless, later in gestation, there was a change in the diastolic BP pattern between pregnancies with a female or a male fetus, where the presence of a male fetus led to higher diastolic BP readings [57]. Interestingly, a recent meta-analysis including > 100,000 participants reported overall higher systolic and diastolic BP in pregnancies with a male fetus compared to a female fetus, which was not attributed to a fetal birth weight genetic score [58].

Furthermore, a study comparing proliferative capacity of trophoblasts in placental villi from individuals with preeclampsia and normotensive controls, revealed that there was an increase in the percentage of proliferative compared to non-proliferative trophoblast cells in female placental villi from preeclampsia, which was associated with excessive syncytiotrophoblast shedding. No difference was observed between male placentas from preeclampsia compared to normal pregnancies [59]. These differences between male and female placental morphology in preeclampsia, characterised by excessive syncytiotrophoblast shedding and potentially increased cell-free fetal DNA in maternal blood [60], are likely associated with increased maternal inflammation that could lead to poorer pregnancy outcomes.

Some of the mechanisms proposed to explain sex differences in preeclampsia with impaired placentation include lower human chorionic gonadotrophin (hCG) in a male placenta of the first trimester, likely inhibited by the higher progesterone levels present in the male placenta, impacting negatively on implantation [61]. In pregnancy, hCG also has an important role in utero-placental angiogenesis and maternal immune system regulation [62].

As described above, strong epidemiological evidence exists between preeclampsia and future increased risk of cardio-metabolic diseases, however the mechanisms of this association are poorly understood. Some of the overlapping signalling pathways reported include those related to inflammation [(IL-6, IL-1, IL-8, C-reactive protein (CRP), monocyte chemoattractant protein-1 (MCP-1) and TNF-α], metabolism [dipeptidyl peptidase 4, insulin growth factor (IGF)1, IGFBP-1, insulin, lipocalin 2, leptin] and angiogenesis/vascular remodelling (TGF-β, Galectin-3, VEGF, endoglin, collagen group proteins and MMPs) [33] . Although there is plethora of evidence to suggest that fetal sex plays an important role in pregnancy and HDP, the importance of sex differences continues beyond pregnancy. As pregnancy is a full challenge for the female body, these underlying cardiovascular risks could be manifested through the occurrence of HDP. Pregnancy also represents a good window of opportunity for intervention and prevention of both short- and long-term cardiovascular disease. Therefore, developing and implementing personalised sex-specific fetal monitoring and treatment strategies for preeclampsia will ensure safe delivery of the baby and prevent death and future morbidities in both mothers and their offspring.

### Acknowledgment of Funding

L.M. acknowledges research support from the Future Leader Fellowship, Level 1, from the National Heart Foundation of Australia (106628).

## C. Obesity, type 2 diabetes, and cardiometabolic diseases

### Alexandra Kautzky-Willer, MD

The prevalence of both obesity and type 2 diabetes mellitus (T2DM) is dramatically increasing worldwide in men and women, but men are usually diagnosed at a lower body fat mass and at younger age than women [63]. This may be caused by the greater visceral and liver fat mass and lower peripheral insulin sensitivity in males compared to females [64]. Thus, overall more men than women are diagnosed with diabetes, although obesity is more common in women at least in some cultures and regions [65]. The presence of two X chromosomes has been associated with greater adiposity, possibly through enhanced expression of genes involved in weight gain which escape X chromosome inactivation [66]. Menopause is linked to increased risk of weight gain due to a decrease of basal metabolic rate and energy expenditure and increased appetite associated with the loss of estrogen. BMI may underestimate body fat mass especially in postmenopausal women [67]; thus, a body composition phenotype defined as osteosarcopenic obesity affects up to 40% of postmenopausal women [68]. Sex differences in phenotypes and clinical outcomes of obesity and diabetes are caused by genetic, epigenetic, and hormonal influences in pathophysiology, clinical manifestation, diagnosis, and response to therapy. Across their lifetime, women experience greater cardiometabolic burden resulting from variations in sex hormones, body fat distribution, and events related to reproduction [69]. Moreover, gender differences arising from psycho-sociocultural processes and environment, such as different behaviours, lifestyles (especially nutrition and physical activity), and attitudes towards prevention and therapy, also impact the development and progression of both obesity and T2DM. In addition, the lifelong continuous interactions between biology and environment start in utero, finally resulting in clinical differences between boys and girls and men and women. Both fetal malnutrition and excess nutrition impact the risk of obesity and T2DM and their complications in later life of the offspring with sexually dimorphic effects [65, 70].

Women appear to be protected from cardiometabolic disease by their sex hormones until menopause, but with the loss of estrogen their risk of disturbances in glucose and lipid metabolism increase, together with a higher risk of subclinical inflammation leading to an increase in cardiovascular risk factors [71]. Women also bear a greater risk factor burden at the time of diagnosis of prediabetes or diabetes compared to men, especially obesity and hypertension [63, 69].  Moreover, socioeconomic factors, education, and psychosocial stress might play a more prominent role in diabetes risk in women. Pregnancies can unmask preexisting subclinical metabolic abnormalities, leading to diagnosis of gestational diabetes, which is the most prominent risk factor for progression to T2DM in women. In addition, the number of women with undiagnosed or diagnosed T2DM at reproductive age is increasing. Pregnancy planning and prepregnancy care is challenging in women with pregestational diabetes putting the women and their offspring at jeopardy of acute and long-term complications.

Cardiovascular complications are the leading cause of death in people with obesity and T2DM with important differences between men and women: Although the absolute risk for cardiovascular mortality is higher in men with diabetes, its relative risk is much greater in women with diabetes [63, 72, 73]. However more recent studies suggest comparable cardiovascular risk between both sexes [74], which is also supported by a recent mendelian randomisation analysis [75]. Atherosclerotic risk increases during menopausal transition associated with increases in insulin resistance, inflammation, endothelial dysfunction, dyslipidemia, and blood pressure and often further weight gain [69]. Unfortunately cardiovascular risk is often underestimated in women with diabetes even in presence of additional risk factors [76]. Moreover women remain undertreated and less often attain their target values of important risk factors like LDL cholesterol, blood pressure, or HbA1c in many studies [63, 77]. Another problem is that they are still underrepresented in clinical trials on cardiovascular risk (CVOTs) and thus important results may be less evident for women. Although the cardiometabolic-renal benefits of new drug classes like SGLT2 inhibitors, GLP-1 receptor analogues, or dual agonists appear to be present in both sexes [78], they are less often prescribed in women even if treatment is recommended according to guidelines because of concomitent heart failure, cardiovascular disease, or chronic kidney disease [79]. Heart failure especially with preserved ejection fraction is much more common in women with obesity or diabetes compared to their male counterparts [80]; thus, SGLT2 inhibitors can clearly reduce disease burden and improve quality of life particularly in older women.

Of note, recent studies reveal that incretin mimetics induce even greater weight loss in women than men [81]. This could be ascribed to the effect of these drugs on reduction of emotional eating, but also other yet unknown sex specific biological factors could play a role.

In summary, there is evidence of numerous clinically relevant sex differences in obesity and diabetes; however, significant gaps remain in our understanding of the underlying pathophysiological mechanisms, hindering the development of sex-specific approaches to precision disease prevention and therapy.

## D. Effects of biological sex in immunological aspects of cardiac disease

### Georgios Kararigas, PhD

There is a growing body of data showing that immune responses play a major role in the development of cardiac disease. In fact, persistent inflammation is known to be detrimental to the heart [82–84]  and the inflammatory response might promote heart failure (HF) [85–88]. Notably, activation of the immune response and pro-inflammatory factrso lead to the inhibition of cardiac contractility [82, 83] . Impaired contractile function of the heart is a major risk factor for HF and sudden death. Importantly, the release of inflammatory factors from one point reaching another can lead to a systemic inflammatory state, thereby resulting in widespread dysfunction and/or injury in adjacent and distant organs, which is common to several cardiometabolic diseases and is also thought to underlie male-biased cardiovascular complications in COVID-19 [89–91].

In this context, significant differences between premenopausal women and men in inflammatory markers have been reported, while these differences were attenuated following menopause and in the absence of hormone therapy [92]. Of even greater relevance, significant sex differences in the transcriptomic regulation of inflammatory genes and pathways in cardiac disease have been documented [93, 94]. In particular, in human pressure overload-induced left ventricular hypertrophy, it was shown that distinct molecular processes are regulated between men and women and that maladaptive cardiac remodeling occurring more frequently in men is associated with greater activation of inflammatory markers [94]. Along this line, analysis of human cardiomyocyte-specific gene regulation revealed that two inflammation-related genes were negatively related to cardiac function as assessed by ejection fraction, with this effect being male specific [93] .

In agreement with those findings in humans, studies employing experimental mice under pressure overload conditions applying the transverse aortic constriction (TAC) method have shown, among others, sex-biased regulation of inflammatory genes and pathways [95–97]. Interestingly, characterization of the transcriptomic response of the heart to pressure overload in female mice lacking estrogen receptor (ER) β showed an increase in inflammatory genes and pathways, such as natural killer cell-mediated cytotoxicity and leukocyte transendothelial migration pathways [96], indicating that the sex steroid estrogen, along with its ERβ, may play an important role in the strict regulation of the inflammatory response.

To this end, menopause-related estrogen decline, which is thought to contribute to the development of HF with preserved ejection fraction (HFpEF) and target organ damage [98, 99], is associated with elevated circulating inflammatory markers, such as tumor necrosis factor (TNF) α, interleukin 6 (IL-6) and plasminogen activator inhibitor-1 [100, 101]. Several of these inflammatory mediators, particularly the plasminogen system, have been implicated as common risk factors for COVID-19 susceptibility [102]. In experimental animals, the removal of estrogen through primarily ovariectomy is also associated with increased levels of inflammation, while exogenous administration of estrogen attenuates these effects, thereby leading to decreased levels of circulatory cytokines, such as TNFα, IL-1β and IL-10 [103–105]. At the molecular level, estrogen exerts a repressive effect on the activity of nuclear factor kappa B (NFκB) by inhibiting its DNA binding ability, thereby down-regulating the activation of NFκB target genes, including TNFα and IL-6 [106, 107]. In addition, estrogen contributes to higher levels of high-density lipoprotein cholesterol and lower levels of low-density lipoprotein cholesterol [108, 109], which might exert an anti-inflammatory effect.

### Acknowledgment of funding

G.K. acknowledges lab support provided by grants from the Icelandic Research Fund (217946-051), Icelandic Cancer Society Research Fund, and University of Iceland Research Fund.  G.K. ORCiD = 0000-0002-8187-0176. 

## E. Sex differences in cardiovascular risk with chronic kidney disease

### Sofia B. Ahmed, MD, MMSc

Cardiovascular-kidney-metabolic (CKM) health describes the closely intertwined relationships across metabolic risk factors, chronic kidney disease (CKD), and the cardiovascular system. Poor CKM health results in widespread pathophysiological effects, most notably the heightened risk of cardiovascular events and associated mortality. Kidney disease is a global epidemic [110].  As recently outlined by the American Heart Association, individuals living with CKD are amongst the highest risk populations for cardiovascular disease and mortality [111], with the risk increasing exponentially with CKD progression [112]. Of note, sex differences in cardiovascular disease (CVD) in CKM syndrome have been highlighted as a major gap in the scientific understanding of mechanisms of CVD development in CKM [113].

#### Sexual dimorphism in kidney structure and function

In general, male kidneys tend to be larger and heavier than female kidneys, with hypertrophy of the proximal tubules, a higher mitochondrial content, greater total nephron count, and distinct transporter expression [114, 115]. In contrast, the glomeruli are notably larger in female children compared to age-matched male children [116].

Animal studies reveal that female kidneys excrete similar amounts of urinary sodium, but at a lower arterial pressure than males [117, 118]. The relative abundance of renal tubular transporters differ by sex [119] which could potentially influence susceptibility to nephrotoxic exposures. For example, numbers of the primary transporter in the proximal renal tubule, the sodium/hydrogen exchanger 3, are lower in females compared to males. In contrast, the abundance of the sodium/chloride co-transporter and epithelial sodium channel are higher in the distal segments of the renal tubule in female rats compared to males [120].

Sex hormones may have a significant impact on kidney development, potentially influencing long-term kidney health [121–124]. For example, in female mice administered testosterone, the kidneys were of increased weight, mainly due to cortical thickening caused by hypertrophy in the glomeruli and convoluted tubules [124]. In nephrectomized rats, male remnant kidneys exhibited a much higher growth rate compared to that of female rats [121]. Although there may be a role for testosterone, monitoring renal mesangial cell proliferation showed no notable effect of testosterone, while estrogen had a modest impact on cell proliferation and reduced overall collagen synthesis [122] .

#### Sex differences in kidney disease

Studies of healthy populations suggest that men have faster age-related loss of estimated glomerular filtration rate (eGFR) than do women. However, observational studies have shown that while earlier stages of CKD (e.g., stage 1–2) are more common in males, primarily as a result of greater albuminuria, more advanced CKD (e.g., stages 3–5) is more prevalent in females overall [125]. Female individuals experience slower CKD progression compared to males [126], although this may be restricted to the premenopausal period [127]. Other studies suggest that neither female sex nor menopause is linked to any significant advantages or risks with respect to CKD progression [128]. These conflicting results likely reflect differences in definitions of outcomes (*e.g*., receipt of dialysis, loss of estimated glomerular filtration rate (eGFR), or reaching a pre-specified eGFR target), variable incorporation of sex-specific factors (*e.g*., complications of pregnancy, menopausal status, exposure to estrogen or testosterone hormonal therapy) and differences in lifestyle and environmental factors, including dietary and medication adherence, level of physical activity, socioeconomic position, and access to health care.

#### Sex differences in cardiovascular-kidney-metabolic syndrome

Using nationally representative National Health and Nutrition Examination Survey data (1988 to 2018) collected from 33,868 US adults, the sex-specific prevalence of CKM syndrome and sex-specific CKM associations with all-cause mortality were recently assessed [129]. While worsening CKM severity was associated with all-cause mortality for all participants, women, in contrast to men, demonstrated a lower incidence of CKM stage 3, but faced higher mortality risk throughout the range of multisystem CKM dysfunction. These results highlight the need to identify the mechanisms driving the combined cardiovascular, kidney, and metabolic system dysfunctions in order to reduce the potential for growing sex-based disparities in multiorgan disease risk.

#### Sex differences in cardiovascular risk with kidney disease: epidemiology

Sex differences in cardiovascular risk exist even in the pediatric population with CKD. While mortality in children with kidney failure is more than 30-fold higher than that of the general population [130], it is significantly higher in girls compared to boys (hazard ratio 1.36; 95% confidence interval 1.25–1.50), with cardiovascular complications representing the most common causes of death [131]. Despite declining overall mortality rates in children with functioning kidney transplants, the proportion of deaths due to cardiovascular causes remains unchanged and remains approximately 20% higher in girls [132]. Girls with advanced CKD are more prone to developing vascular stiffening than boys, which is in contrast to the physiological development demonstrated in healthy children; interestingly, this is independent of the cause of CKD [133]. Furthermore, these sex differences continue even after receipt of a kidney transplant and may contribute to the higher mortality rates observed in girls with kidney failure [133]. Studies in adult populations indicate that the female survival advantage observed in the general population is lost in the setting of kidney failure, with more years of life lost in female patients, who exhibit more excess deaths from cardiovascular disease irrespective of cause of CKD [134, 135].

A meta-analysis involving almost 100,000 participants with CKD revealed that men were at marginally higher risk of cardiovascular mortality than women among the CKD population, with borderline significance [136]. A pooled analysis of more than 2 million individuals revealed that men had higher cardiovascular and all-cause mortality across all levels of estimated glomerular filtration rate (eGFR), though the risk of cardiovascular death increased more sharply in women as eGFR declined [128]. In the early stages of CKD, women face a lower cardiovascular risk than men, but this difference diminishes at lower eGFR levels [137]. For example, a Swedish study involving 30,000 CKD patients in stages 3–5 reported that cardiovascular mortality was 20% higher in men than in women, but no sex differences were observed in stage 5 CKD non-dialysis-dependent patients [138]  These findings suggest that the protective effect of female sex on cardiovascular health diminishes as CKD progresses [136, 139–141]. It has been speculated that in women, more severe microvascular disease (rather than macrovascular disease), may contribute to the interaction between CKD stage and atheromatous and non-atheromatous outcomes [141]. Insulin resistance is a greater cardiovascular risk factor in women compared to men [142]. Although males are overall at higher CVD risk than females, this association is attenuated or even reversed in the setting of diabetes [143, 144]. Men are more likely to demonstrate impaired fasting glucose, while impaired glucose tolerance is more common among women [145]; whether this contributes to poorer cardiovascular outcomes in women is not clear.

Hypertension contributes not only to the development and progression of CKD but also to cardiovascular risk in both men and women [146].   Blood pressure is higher in men than women, although an accelerated age-related rise in blood pressure begins around the fifth decade of life in women [147]. Of note, increasing cardiovascular disease risk begins at lower thresholds of SBP for women than for men [148]. Salt-sensitive blood pressure, a cardiovascular risk factor, is more prevalent in women than in men, including during premenopause [149]. In an observational study of over 4000 participants, women demonstrated greater SBP changes compared to men in the setting of multiple types of metabolic stress (including decrements of eGFR), particularly in periods of transition from metabolic health to disease [150].

Gender-related factors may also play a role in sex differences in cardiovascular risk observed in men compared to women with CKD [151]. While men are more frequently prescribed angiotensin-converting enzyme inhibitors and statins [152, 153 , they also exhibit higher rates of behavioral cardiovascular risk factors that influence risk of CKD, such as smoking and alcohol consumption, and are more likely to have poorer dietary habits. In contrast, women tend to adopt primary cardiovascular prevention strategies more readily than men [154]. Achievement of targets for cardiovascular risk factors including blood pressure control and LDL cholesterol control are reported to be less common in females than in males in the setting of CKD and diabetes [153, 155, 156].

#### Sex differences in cardiovascular pharmacologic therapy in kidney disease

There is a higher risk of adverse drug events with use of cardiovascular medications in women than men, which is likely due to sex-based differences in the absorption, distribution, metabolism, and excretion of drugs [157]; of note, these differences in pharmacokinetics and pharmacodynamics may be exacerbated in the setting of kidney disease [158, 159]. Pre-clinical research has suggested that sex-based differences in the renin–angiotensin–aldosterone [160–162] and endothelin systems [163, 164] may influence the safety and efficacy of medications commonly used for treatment of both cardiovascular and kidney disease. This has borne out in humans: while the selective type-A endothelin receptor antagonist atrasentan slowed progression of CKD in individuals with type 2 diabetes, there was greater kidney protection in female than in male participants, but also more heart failure events in female than in male participants [165]. In a *post-hoc* analysis of the Angiotensin II Antagonist Losartan Study and Irbesartan type II Diabetic Nephropathy Trial results examining the effects of angiotensin receptor blockers (ARBs) on kidney outcomes in participants with type 2 diabetes, the beneficial effects of ARBs were similar in male and female participants for the kidney outcome, but cardiovascular risk was only lowered in male but not in female participants [166]. These data suggest that sex-specific dosing regimens may be considered to optimize cardiovascular treatment in the setting of CKD.

#### The impact of sex hormones on cardiovascular risk in kidney disease

The kidney plays a critical role in regulating sex hormones [167]. In individuals with CKD, there is a significant disruption of the hypothalamic–pituitary–testicular axis, which appears to become more pronounced as kidney function deteriorates. The hormonal profile in women with CKD typically includes elevated levels of LH and PRL, reduced AMH, and a substantial decrease in serum estrogen [168]. Men with CKD have similar disturbances in hormonal profile, although with a reduced serum concentration of testosterone rather than estrogen [169].

Two systematic reviews and meta-analyses investigating links between sex hormones and the risk of cardiovascular disease and mortality reported that lower total testosterone concentrations were associated with an increased risk of cardiovascular events and all-cause mortality in men with CKD [170, 171]. Due to a lack of published data, the authors were not able to comment on the relationship between testosterone and these outcomes in women with CKD [171]. The impact of testosterone supplementation on cardiovascular outcomes in persons with CKD is unknown.

Two studies have explored the connection between circulating estradiol concentrations and the risk of cardiovascular and all-cause mortality in women with CKD. One study reported a U-shaped relationship between serum estradiol concentrations and the risk of cardiovascular [HR 5.13 (1.29–20.3) and 4.21 (1.17–15.1)] and all-cause mortality [HR 4.49 (1.59–12.6) and 4.32 (1.59–11.7)] in postmenopausal women undergoing hemodialysis, with those in the lowest and highest serum estradiol concentration tertiles showing the highest risk [172].  Another cohort study reported that higher estradiol levels were associated with an increased risk of all-cause, but not cardiovascular mortality in premenopausal and postmenopausal-aged women with kidney failure undergoing hemodialysis [HR 1.86 (1.14–3.01) [173]. A systematic review on the effects of postmenopausal hormone therapy on cardiovascular outcomes in women with CKD indicated that while no studies have included cardiovascular events or mortality, hormone therapy was associated with increased HDL cholesterol and decreased LDL cholesterol levels [174].

## E. Sexual dimorphism in the cardiovascular and metabolic effects of the androgens

### John N. Stallone, PhD, FAPS

#### Introduction

The male sex steroid hormones (testosterone (TES) and related C19 androgen molecules) are the humoral messengers responsible for the differentiation, development, and maintenance of the male reproductive system and secondary sexual characteristics that differ so markedly between males and females. Although the anatomical, behavioral, and reproductive effects of TES were recognized in animal experiments as early as the eighteenth and nineteenth century (long before its chemical structure as the principal mammalian male sex hormone of testicular origin was identified in 1935) [175, 176], it is now clearly recognized that the androgens exert a broad variety of physiologically relevant effects on the regulation of cardiovascular, hematopoietic, immune, metabolic, and nervous systems as well as their well-known anabolic effects on bone and skeletal muscle [177, 178]. It is important to recognize that the androgens are synthesized in both sexes and circulate in the plasma, albeit at quite different levels. Plasma levels of TES in females are 5–10% of those in males; however it is increasingly recognized that the androgens play unique roles in the regulation of reproductive as well as cardiovascular, metabolic, and other functions in females as well as males [177, 178].

#### Sex differences in the cardiovascular effects of androgens

Cardiovascular diseases (CVD) are a major cause of morbidity and mortality and in the Western world, one-third of all deaths are attributable to CVD [179]. A conspicuous feature of this healthcare epidemic is that most forms of CVD are higher in men than in premenopausal women, yet the reasons for these prominent sex differences remain unclear. The clinical case studies and epidemiological observations that hypertension (HT) and coronary artery disease (CAD) occur more frequently in men than in premenopausal women [180–186] have led to the dogmatic view that TES and other androgens exert deleterious effects on the heart and vasculature and worsen the development of CVD in men, in part by exacerbating risk factors such as blood pressure (BP), body fat composition, insulin resistance, and serum lipid profiles [187–189]. In parallel, earlier animal studies provided support for this dogma and revealed that in various rat models of HT castration attenuates the development of HT in males [190–194]. However, more recent human clinical trials [195–197] and experimental animal studies [198–201]  reveal that TES and its metabolites exert beneficial effects on BP and metabolic function in males, which are risk factors for CVD. Reconciliation of the conflict between earlier and more recent studies on the cardiovascular effects of TES depends upon careful scrutiny of earlier experimental animal and human studies which suffered from flaws or limitations in experimental design and/or the animal models employed [184, 186, 196]. For example, most earlier animal studies employed unrealistic models and short term settings. Similarly, subsequent analyses of earlier human clinical and epidemiological studies revealed important validity issues with experimental design, data collection and analysis, and selective exclusion of data, which emphasize the importance of careful study design and that dogma and controversy can adversely distort the validity of human clinical and epidemiological findings concerning TES (for detailed review of these issues, see [202]).

Human clinical and experimental animal studies have clearly established that TES and other androgen metabolites exert beneficial effects by inducing relaxation of vascular smooth muscle (VSM) through rapid, nongenomic (androgen receptor (AR)-independent) mechanisms in vitro (for recent reviews, see [203–205]. Although this acute effect of TES and other androgens was initially reported at high (micromolar) concentrations in variety of large arteries from several species, more recent studies revealed that TES produced relaxation of smaller resistance arteries and arterioles at nanomolar (physiological) concentrations (*i.e.,* mesenteric, prostatic, pulmonary, and subcutaneous) (for reviews, see [202–205]. The key mechanism underlying this effect of TES on VSM appears to be activation of calcium-dependent (BK_Ca_) and voltage-operated (K_V_) K^+^ channels via TES-induced activation of neuronal nitric oxide synthase (nNOS) and/or inactivation of L-type voltage-operated Ca2^+^ channels in VSM [204, 205]. Although numerous studies have clearly established that TES and other androgens exert rapid, nongenomic vasorelaxing effects in vitro*,* evidence that TES produces coronary or systemic vasodilation in vivo at physiological concentrations (100 pM to 100 nM) is more limited. Studies in anesthetized dogs [206], pigs [207] and humans [208] demonstrated that intra-arterial infusions of TES produces coronary vasodilation, and regional vasodilation of mesenteric, renal, and skeletal muscle vascular beds in anesthetized pigs [207]. More recently, several studies reported that TES and other androgens produced systemic vasodilation. In conscious, ganglionic-blocked male Sprague–Dawley (SD) rats, *i.v.* infusion of TES or its genomically inactive metabolite 5β-dihydrotestosterone (5β-DHT) produced dose-dependent systemic hypotension [198]. Similarly, bolus *i.v.* injections of TES, 5β-DHT, and the potent genomically active metabolite 5α-DHT produced dose-dependent hypotension in Spontaneously Hypertensive (SHR) and to a lesser extent in normotensive-control WKY male rats [199]. In both studies, the hypotensive effect of 5β-DHT was more efficacious than that of TES. While these recent studies clearly reveal that exogenous TES and its metabolites exert important hypotensive effects on systemic BP through direct vasodilatory actions on the systemic vasculature, the role of endogenous androgens in the long-term regulation of BP remained unanswered until recently. Long-term studies by Perusquia et al. [200] revealed that castration of both Wistar and WKY male rats led to the progressive development of HT over a period of 11 weeks that then plateaued through 18 weeks (151 ± 2 *vs.* 110 ± 2 mmHg at baseline, mean arterial BP). Subsequent long-term studies by Hanson et al. [201]. demonstrated that castration of male SD rats produced progressive HT from 109 ± 3 at baseline to 143 ± 3 mmHg systolic BP at 10 weeks, and that subsequent TES replacement therapy to physiological levels completely normalized BP in 5 weeks to 113 ± 1.3 mmHg. Interestingly, nearly identical effects of castration and TES replacement were observed in AR-deficient Testicular-feminized male (Tfm) rats, strongly suggesting that the cardiovascular effects of TES are nongenomic in nature. Treatment of SD rats with the type 1 angiotensin receptor antagonist Losartan completely prevented development of HT. rt-PCR of the kidney revealed that castration increased expression of mRNA for renin (92%), angiotensin converting enzyme (58%), and angiotensin type 1 receptor (80%), while TES replacement therapy completely normalized renin-angiotensin system (RAS) mRNA expression to levels of intact control male rats. These findings reveal that both endogenous and exogenous TES exert anti-hypertensive effects that appear to involve reductions in RAS expression in the kidney, enhanced fluid excretion, and enhanced systemic vasodilation.

The overwhelming majority of studies on the cardiovascular effects of the androgens in experimental animals, and to a lesser extent in human clinical trials, have employed males. However, given that measurable levels of TES and other androgens are present in the circulation of female animals and humans, then it is also important that the effects of these hormones be studied in females. Studies of the acute effects of TES on blood vessels isolated from females, while limited in number, have uniformly revealed that TES-induces vasorelaxation of rat aorta, human pulmonary artery and vein, and isolated, perfused human lung do not differ between males and females [209–211]. Similarly, acute intra-arterial infusions of TES in anesthetized pigs produced similar regional vasodilation in females as in males [207]. In near-term normal-pregnant and preeclamptic-pregnant female Wistar rats, bolus *i.v.* injections of TES, 5α-DHT, 5β-DHT, and dehydroepiandrosterone (DHEA) produced similar substantial reductions in mean arterial BP in both groups, while 5β-DHT and DHEA exhibited significantly greater hypotensive potency than TES or 5α-DHT [212]. Isolated thoracic aortae from these same pregnant groups also exhibited similar vasorelaxing responses to these same androgens. With regard to long-term effects of TES treatment on vascular function and BP, several studies have examined effects in female animals and humans. Long-term treatment of female Cynomologous monkeys with TES while fed a high fat diet increased coronary arterial atherosclerotic plaques but improved vasomotor responses to acetylcholine [213].  In contrast, long-term treatment of ovariectomized female SHR rats with TES for 5–10 weeks increased arterial BP to levels similar to those of male SHR [190, 214]. Although not a goal of this study, this experimental design served as a model to test the effects of cross-sex hormone replacement in female-to-male transexuals, and resulted in similar detrimental effects as long-term TES treatment in human female-to-male transexuals, which impaired flow-mediated vasodilation [215]. Similarly, several prospective human clinical trials identified positive relationships between plasma free (bioavailable) TES levels and incidences of elevated BP, HT, and CAD in pre- and post-menopausal women [216–218]. While these human studies appear to conflict with the majority of experimental animal studies on the vascular effects of TES, the former studies could not discern whether elevations in plasma TES followed or preceded the increases in BP, HT, and CAD in human females. Likewise, long-term treatment of females with doses of TES that elevated plasma levels to those of males likely produce very different cardiovascular effects than the much lower normal plasma levels of TES in females. Indeed this possibility in quite likely since male levels of TES in female animals are associated with detrimental metabolic effects, while the same levels in males produce beneficial metabolic effects (for detailed review of these issues, see [202]). In summary then, while acute vascular effects of TES and other androgens are clearly beneficial and similar in males and females, long-term systemic effects of the androgens may differ in males and females and are likely related to the pronounced sex differences in plasma levels of TES, as well as the distribution and numbers of receptors present in the target tissues.

#### Sex differences in the metabolic effects of androgens

The early eighteenth and nineteenth century experiments of Hunter and Berthold on roosters [175, 176] and those of Brown-Sequard involving self-injection of aqueous extracts of dog and guinea pig testes (so-called organotherapy) [219] led to the recognition that a humoral substance from the testes (identified much later as TES in 1935) was responsible for beneficial effects not only on reproductive tract function, but also on overall vitality, physical strength, and intellectual capacity. As discussed above, earlier human epidemiological studies and clinical trials led to two key areas of study in the 1960s that continue today, the effects of androgens on cardiovascular and metabolic health and disease. An extensive review of these earlier studies led Kalin and Zumoff [180] to conclude that androgens exerted direct detrimental effects on the atherogenic process in the coronary arteries, contributing to the development of CAD. This view reflects the long-known major influence of androgens on body fat composition, muscle mass, and bone density in the male [220, 221] and the idea that these hormones exerted detrimental effects on lipid metabolism (*e.g*., dyslipidemia), an important risk factor for CVD. This belief was furthered by clinical observations that the use of anabolic steroids by athletes (synthetic derivatives of TES) to enhance muscular strength resulted in premature, higher incidences of HT, ventricular remodeling, and sudden cardiac death [222–224].  However, more recent human epidemiological studies and clinical trials have challenged this dogmatic view and revealed that in addition to the well-known classical effects of TES on bone density and muscle mass, that it also plays a key beneficial role in the regulation of carbohydrate, fat, and protein metabolism and inflammation, and that TES deficiency is strongly associated with increased fat mass, hyperlipidemia, hypercholesterolemia, hyperglycemia, and insulin resistance, as well as elevated BP, the cluster of symptoms comprising metabolic syndrome (MetS) and type 2 diabetes mellitus (T2DM). MetS and T2DM share a common etiology (central adiposity) and are central risk factors for CVD (for reviews see [202, 220, 221, 225]).   Indeed, the link between hypogonadism and the development of visceral obesity, insulin resistance, and MetS in men has been well established by many recent studies (for review, see [226]).  

Human clinical and experimental animal studies have identified several key actions of TES in the male that promote glucose and lipid homeostasis, including: prevention of visceral fat accumulation (adipogenesis), improved insulin sensitivity in adipose, skeletal muscle, liver, and brain, central (hypothalamic) effects to enhance energy expenditure and leptin sensitivity, and regulation of pancreatic β-cell function to improve glucose tolerance and glucose-stimulated insulin secretion. These findings have led to a proposed mechanism of androgen actions to promote glucose and energy homeostasis via AR-mediated effects on adipose tissue, liver, pancreatic β-cells, skeletal muscle, and metabolic centers in the hypothalamus [226]. Interestingly, aromatization of TES to 17β-estradiol that interacts with the estrogen receptor (ER) appears to be at least partly responsible for preventing abdominal obesity; thus, both TES and estrogen appear to play important roles in the regulation of energy homeostasis in males.

The dramatic sexual dimorphism in the metabolic effects of androgens in men *vs.* women is reflected by the striking differences in body fat distribution and skeletal muscle mass observed between the sexes, which begin at birth and are enhanced greatly at puberty with the surges of sex steroid hormone secretion. Thus, men tend to have less total body fat but more abdominal adipose tissue (*i.e.,* android distribution) and greater skeletal muscle mass, driven by the effects of TES. In contrast, women tend to have more total body fat distributed with a gluteal/femoral and subcutaneous (*i.e.,* gynoid) distribution and less skeletal muscle mass, driven by the metabolic actions of estrogen [227–229]. While estrogen in the female mediates the amount, deposition, and function of the metabolically safer gynoid body fat distribution, TES on the other hand drives the deposition and function of the more unfavorable abdominal adipose tissue in the male; however, this is metabolically compensated for by the anabolic effects of TES to increase lean tissue and skeletal muscle mass and function to impair adipogenesis.

While the role of TES in the regulation of metabolism in men and the impact of TES deficiency on the development of visceral obesity, insulin resistance, and the MetS is well established, the the role of androgens in the regulation of metabolism in females and the possible impact of abnormalities in TES levels on metabolic dysfunction in women has not been well studied [230], even though associations between androgen excess and diabetes, obesity, and infertility have been known for nearly a century. Indeed, the relationship between androgen excess and diabetes has been known since the report of “diabetes in bearded women” by Achard and Thiers in 1921 [231]. Similarly, the link between obesity and the clinical triad of polycystic ovaries, hirsutism, and oligo/amenorrhea was first reported in 1935 as the Stein-Leventhal Syndrom [232], which was later renamed as polycystic ovary syndrome (PCOS). Thus, exactly opposite of the situation in males, the coexistence of excess androgen levels with cardiovascular risk factors (*i.e.,* dyslipidemia, insulin resistance, and obesity) and increased atherosclerosis [233] that occur in PCOS has advanced the concept that excess androgens exert adverse effects in women [234, 235]. 

Likewise, other hyperandrogenic conditions in women such as congenital adrenal hyperplasia (CAH) and androgenized female-to-male transexuals are also associated with metabolic dysfunction, especially glucose intolerance, insulin resistance, obesity, and subsequently with T2DM [226]. Thus, the much lower levels of TES in normal women appear to exert beneficial effects, while the elevated androgen levels in pathophysiological states such as PCOS result in cardiovascular and metabolic dysfunction; however, this sexual dimorphism in the metabolic effects of androgens in males *vs.* females is puzzling and poorly understood [236, 237]. The aforementioned observations have led to the concept that a delicate equilibrium exists between androgen effects on adipose tissue *vs.* skeletal muscle that underpins the metabolic phenotype seen with androgen excess in females *vs.* androgen deficiency in males. This concept of overlapping adverse metabolic effects of androgen excess in women *vs.* androgen deficiency in men has been termed the “metabolic valley of death” [236] (for more detailed review, see [202]). 

The dramatic sexual dimorphism in the relationship between TES and metabolic function and the delicate balance of androgen effects in adipose *vs.* skeletal muscle raises the question of what mechanism(s) underlie the disruption of the equilibrium in TES effects? Three major factors appear to be involved; namely, gonadal dysfunction, normal aging, and sedentary lifestyle. Thus, in the male, these disturbances lead to a deficiency of testicular androgen secretion, resulting in increased abdominal fat deposition, dyslipidemia, loss of skeletal muscle mass, increased insulin resistance, and development of MetS and T2DM. Conversely, in the female, age-dependent reductions in estrogen and relatively small increases in circulating androgens from PCOS or CAH (or larger increases with cross-sex hormone therapy in female-to-male transexuals) lead not only to phenotypic masculinization, but also cause “masculinization” of adipose tissue and its conversion from gluteal/subcutaneous fat to abdominal (visceral) fat deposition with the expression of pro-inflammatory cytokines similar that observed in males [238, 239].   It has been proposed that the excess TES and AR activation lead to deleterious effects on glucose, fat, and energy homeostasis, including: activation of adipose tissue (increased adiposity and inflammation), central (hypothalamic) effects to reduce energy expenditure and leptin sensitivity, activation of macrophages (oxidative stress), excess pancreatic β-cell function (insulin secretion), and skeletal muscle insensitivity to insulin. Together, these effects synergize to promote metabolic dysfunction, inflammation, visceral adiposity, and eventually, T2DM [226]. 

In summary then, the available data from both experimental animal and human studies reveal the existence of a bi-directional modulation of glucose and fat homeostasis in females *vs.* males. Thus, androgen or AR deficiency results in dramatic metabolic dysfunction in aging males, but to a much lesser extent in females. Since AR activation is weaker in females due to (normally) substantially lower circulating androgen levels and a much smaller population of AR in metabolic target tissues, TES and other androgens are less important in the maintenance of energy homeostasis in females under normal conditions; however, the elevated levels of androgens that occur with PCOS, CAH, or other pathological conditions are sufficient to result in metabolic dysfunction. Likewise, the substantial levels of TES resulting from cross-sex hormone therapy to masculinize female-to-male transexuals likely produces significant metabolic dysfunction, increasing the risk factors for the development of HT and CVD, especially MetS and T2DM.

#### Physiological relevance and conclusions

It is increasingly apparent that endogenous TES and other androgens exert widespread beneficial effects on cardiovascular and metabolic functions. Recent human clinical trials over the last 10 years increasingly challenge the long-standing dogma that TES exerts detrimental effects on male cardiovascular and metabolic health and is largely responsible for the greater incidence of CVD in men than in women. Instead, it is now apparent that it is the gradual decline in circulating TES levels that are a normal part of the aging process that contributes to age-dependent increases in CVD and metabolic dysfunction in men. In parallel, recent experimental animal studies reveal that castration of male rats results in long-term development of HT that is completely reversed by physiological TES replacement therapy (TRT). Further, clinical hypogonadism in aging men is associated with both HT and MetS, which exacerbate the development of CVD. While the most recent human clinical trials overwhelming report that TRT does not increase risk of CVD or mortality in older hypogonadal men and is associated with reductions in BP and MetS, these studies do not provide unequivocal evidence that TRT is safe and does not increase risk of cardiovascular events. However, it is also clear that some human studies suffer from poor experimental design and statistical analysis and investigator bias. Thus, unequivocal proof that TRT is safe and beneficial for the treatment of hypogonadism and associated cardiovascular and metabolic dysfunctions in men will require more better designed clinical trials. Similarly, our understanding of the role of androgens in female health and disease is still relatively limited and more studies are needed to elucidate the mechanisms underlying the striking sexual dimorphism in the cardiovascular and metabolic effects of TES.

### Acknowledgment of funding

J.N.S. acknowledges research support from the State of Texas.

## F. Conclusions and significance

The foregoing mini-reviews clearly reveal the striking importance of sex differences in cardiovascular, metabolic, and immune system functions in health and in the pathogenesis of disease processes, which likely involve a combination of effects of the sex chromosomes as well as the gonadal steroid hormones. It is noteworthy that these sex differences begin in the developing fetus, and that fetal sex clearly influences the pathogensis of the hypertensive diseases of pregnancy. Moreover, the differences in the effects of fetal sex continue beyond pregnancy and appears to influence the future risk of maternal cardiometabolic diseases. Indeed, there is strong evidence of many clinically-relevant sexually dimorphic characteristics of obesity and T2DM. Again, there is evidence that both chromosomal and humoral effects underlie the significant sex differences observed in obesity and diabetes; however, the underlying pathophysiological mechanisms are poorly understood and await further clinical and experimental animal studies. The gonadal steroid hormones (both androgens and estrogens) are known to have important effects on the regulation of intermediary metabolism; however, recent studies reveal the emerging importance of these hormones in the regulation of inflammation. Clinical and experimental studies have shown that menopausal declines in estrogen are thought to contribute to the development of heart failure in women, and this has been associated with elevations in circulating inflammatory markers. Similar effects on inflammatory function may also occur in men with the progressive age-dependent declines in testosterone. While the androgens appear to exert beneficial cardiovascular and metabolic effects in men, their effects in women are poorly understood. The much lower levels of testosterone in normal women appear to exert similar effects on the regulation of glucose and lipid metabolism as the much higher levels in men. These effects appear to be bi-directional between the sexes, since pathophysiological increases in androgen levels in women are associated with the same metabolic disturbances observed with age-dependent declines in testosterone in men; namely, hypertension, metabolic syndrome, and type 2 diabetes mellitus. Finally, it is becoming increasingly apparent that the kidney plays an important role in the regulation of sex steroid hormone levels, which appear to decline with chronic kidney disease. The declines in both estrogen and testosterone have been associated with increases in cardiovascular risk factors; thus, these hormones appear to play a central role in the linkage between chronic kidney disease and the subsequent development of cardiovascular disease.

In conclusion, it is clear that sex differences in cardiovascular, metabolic, and immune function play important roles in health and in the pathogenesis of disease. Elucidation of the chromosomal and humoral mechanisms underlying the sexual dimorphism in physiological functions will play an important role in the future development of age- and sex-specific prevention and pharmacotherapy of disease processes.

## Data Availability

No datasets were generated or analysed during the current study.
